# Supplementary material for: Evolutionary genomics of camouflage innovation in the orchid mantis
Source: Nat Commun. 2023 Aug 10;14:4821. doi: 10.1038/s41467-023-40355-1 (PMC10415354; doi:10.1038/s41467-023-40355-1)
Supplement: Supplementary file 1 — Supplementary information [file 41467_2023_40355_MOESM1_ESM.pdf]

Supporting Information for
Evolutionary genomics of camouflage innovation in the orchid mantis

This PDF file includes:

Supplementary Figures 1 to 16
Supplementary Tables 1 to 10

Supplementary Figures and Figure Legends

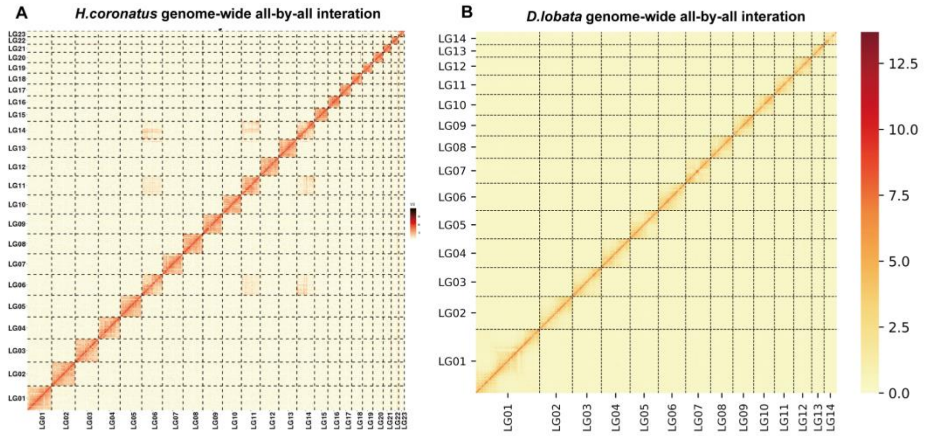

Supplementary Fig. 1 Genome-wide all-by-all interactions of *H. coronatus* (A) and *D. lobata* (B). The heatmap coordinates represent chromosomes, and the color of each point represents the log value of the interaction strength of the corresponding genome bin pair. The mutual strength increases sequentially from yellow to dark red.

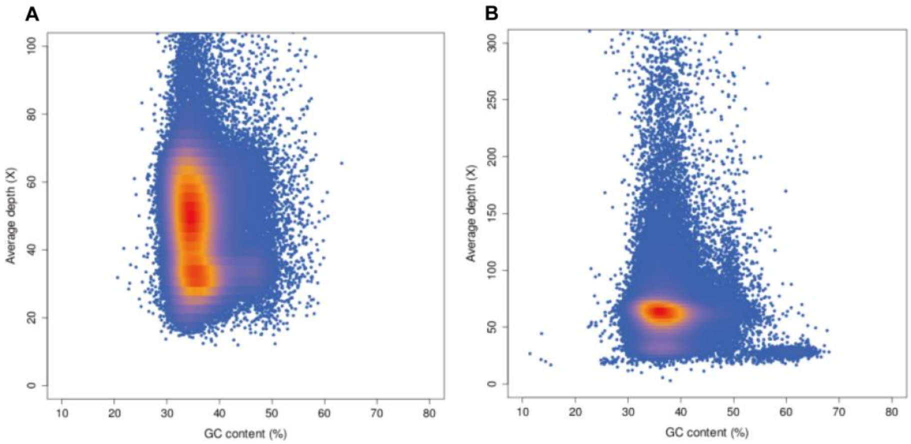

Supplementary Fig. 2 GC depth distribution of *H. coronatus* (A) and *D. lobata* (B). The X-axis is the GC content, and the Y-axis is the average depth. These two values are counted sequentially in a 50 kb window.

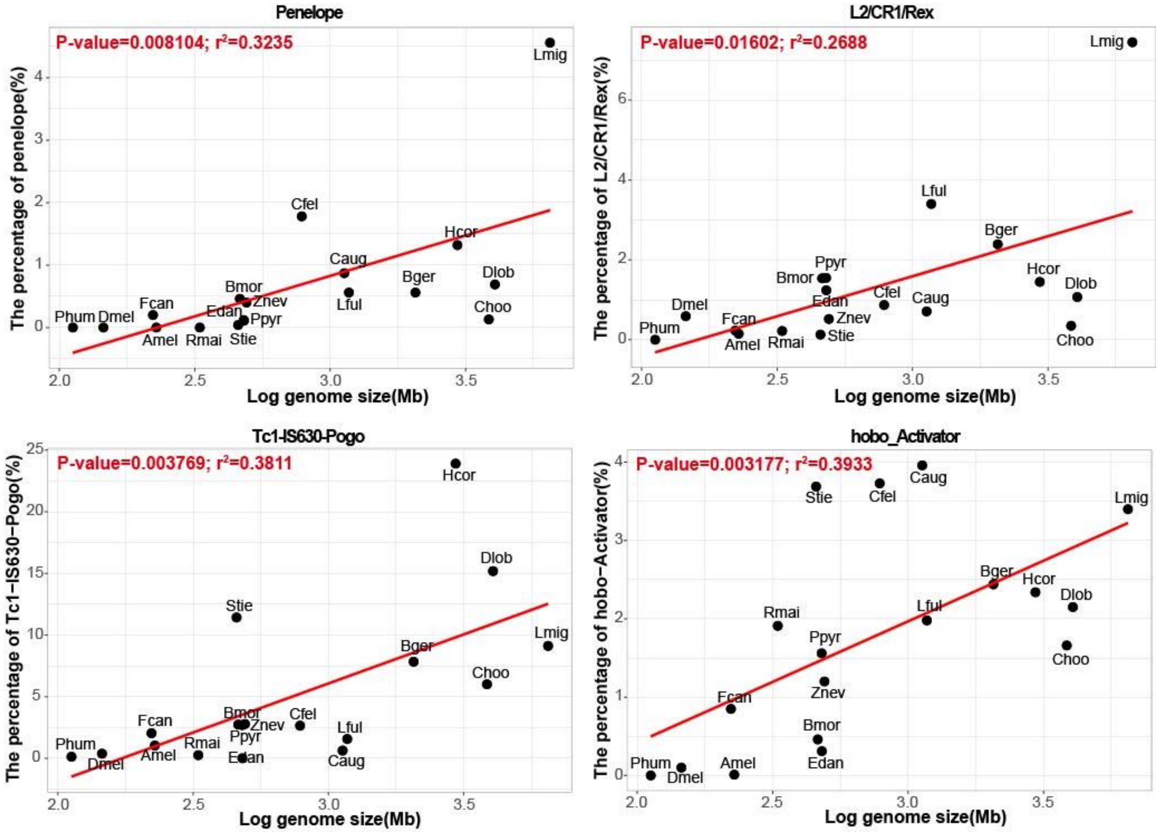

Supplementary Fig. 3 Phylogenetic generalized least squares regression analysis was used to analyze the relationships of DNA transposons (hobo-Activator, Tc1-IS630-Pogo) and retrotransposons (L2/CR1/Rex, Penelope) with insect genome size. The horizontal axis represents the log (genome size), and the ordinate represents the proportion of transposons in the genome. Each point represents a species. Hcor, *Hymenopus coronatus*, Dlob, *Deroplatys lobata*, Znev, *Zootermopsis nevadensis*, Bger, *Blattella germanica*, Dmel, *Drosophila melanogaster*, Lmig, *Locusta migratoria*, Choo, *Clitarchus hookeri*, Bmor, *Bombyx mori*, Amel, *Apis mellifera*, Cfel, *Ctenocephalides felis*, Stie, *Stenopsyche tienmushanensis*, Fcan, *Folsomia candida*, Lful, *Ladona fulva*, Csec, *Cryptotermes secundus*, Phum, *Pediculus humanus*, Ppyr, *Photinus pyralis*, Caug, *Campodea augens*, Rmai, *Rhopalosiphum maidis*.

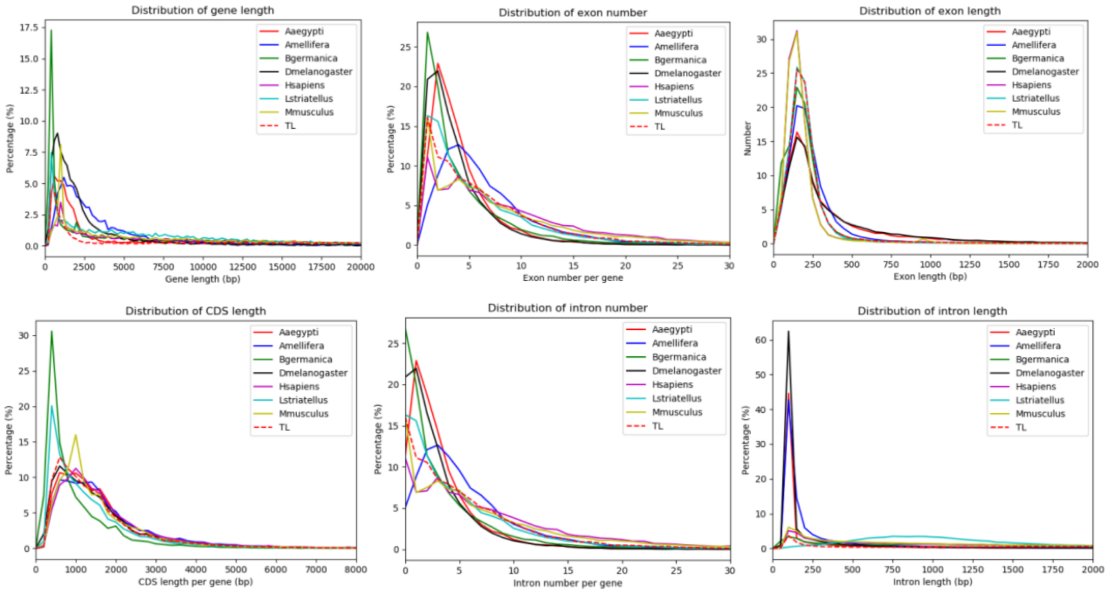

Supplementary Fig. 4 Distribution map of gene elements in the orchid mantis (TL) and 7 other species (*A. aegypti*, *A. mellifera*, *B. germanica*, *D. melanogaster*, *H. sapiens*, *L. striatellus*, and *M. musculus*), including gene length, CDS length, exon number, exon length, intron number and intron length. Different species are represented by different colors.

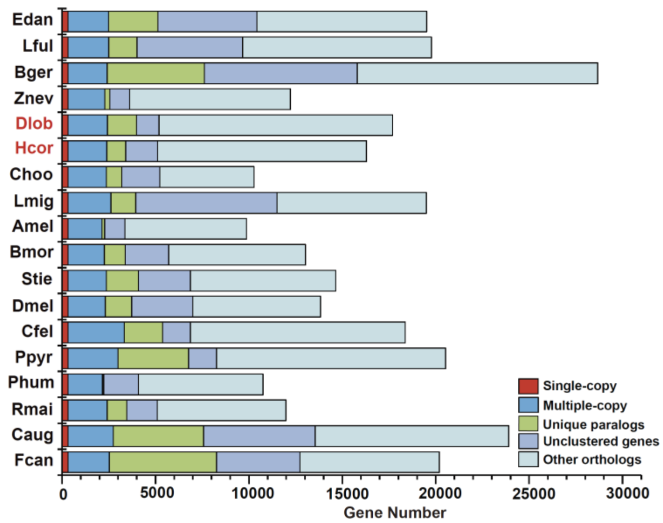

Supplementary Fig. 5 A bar chart showing protein orthology across taxonomic groups within each genome. Different colors represent different orthologs. From left to right represent single copies, multiple copies, unique paralogs, unclustered genes and other orthologs. The vertical axis represents different species, and the horizontal axis represents the gene number. Hcor, *Hymenopus coronatus*. Dlob, *Deroplatys lobata*. Znev, *Zootermopsis nevadensis*. Bger, *Blattella germanica*. Dmel, *Drosophila melanogaster*. Lmig, *Locusta migratoria*. Choo, *Clitarchus hookeri*. Bmor, *Bombyx mori*. Amel, *Apis mellifera*. Cfel, *Ctenocephalides felis*. Stie, *Stenopsyche tienmushanensis*. Fcan, *Folsomia candida*. Lful, *Ladona fulva*. Csec, *Cryptotermes secundus*. Phum, *Pediculus humanus*. Ppyr, *Photinus pyralis*. Caug, *Campodea augens*. Rmai, *Rhopalosiphum maidis*.

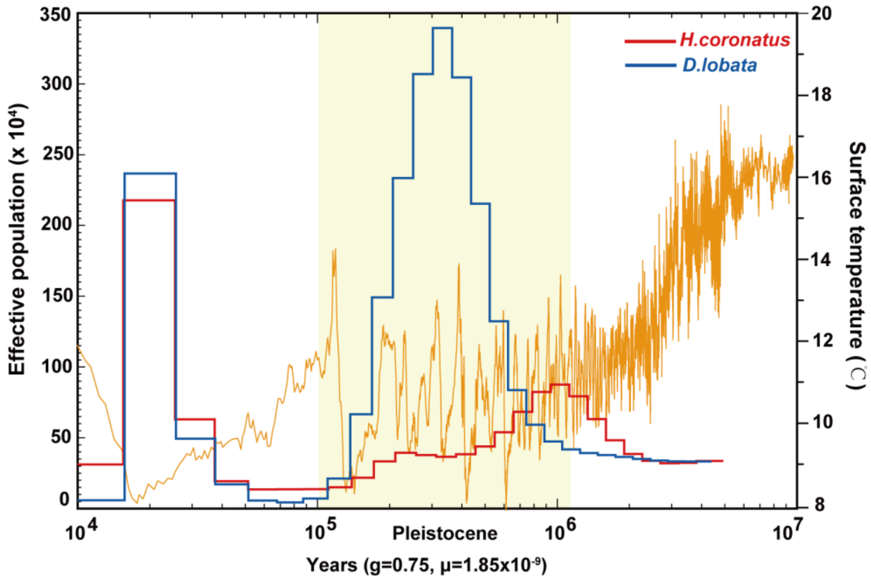

Supplementary Fig. 6 Historical effective population size (Ne) of *H. coronatus* and *D. lobata*. The unit of the x-axis is year. The result is scaled using a generation time (g) of 0.75 years and a per-base mutation rate (μ) of 1.85 × 10^{-9}. The atmospheric surface air temperature (°C) is indicated by the yellow line. The yellow region indicates the Pleistocene period.

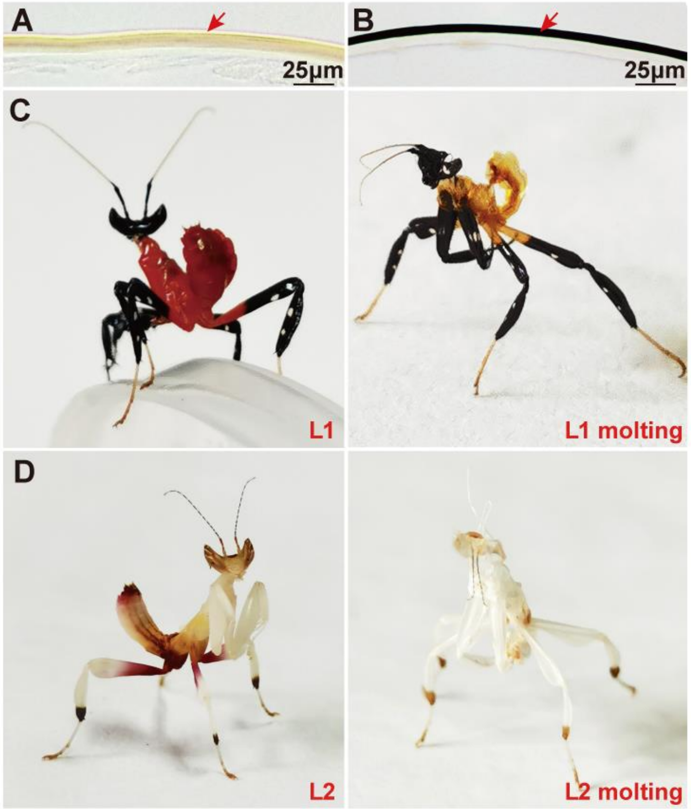

Supplementary Fig. 7 Observation of the red (A) and black (B) areas of the first instar of *H. coronatus* by optical microscopy. The red arrow marks the cuticle of the insect. Scale bar = 25 μm. The individuals (left) and molted epidermis (right) of the first (C) and second (D) instars of *H. coronatus*.

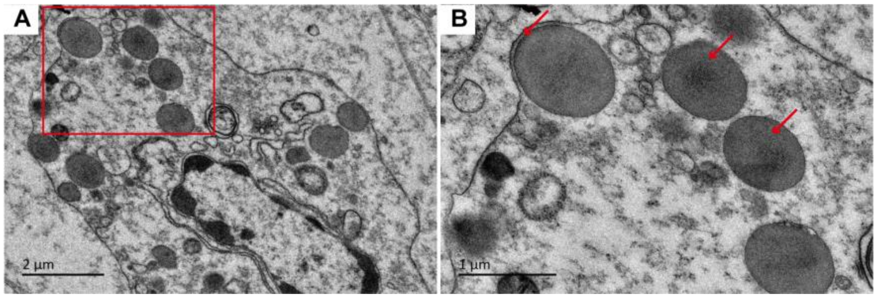

Supplementary Fig. 8 Ultrastructure of the femoral lobes of *H. coronatus*. (A) The mature pigment granules. (B) The magnified area marked by a red box in A. Scale bar = 1 μm. The red arrows highlight pigment particles. The experiment was repeated twice with similar results.

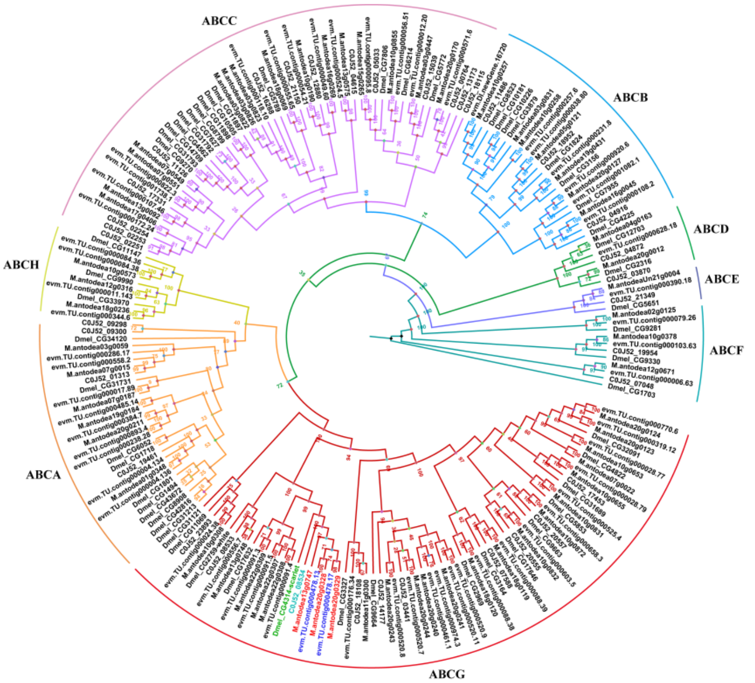

Supplementary Fig. 9 Phylogenetic tree of the ATP-binding cassette (ABC) gene family of *H. coronatus*, *D. lobata*, *B. germanica* and *D. melanogaster*. The maximum likelihood tree was constructed on the basis of multiple alignments of the ABC transporter protein sequences. The colors of internal nodes indicate eight subfamilies (ABCA- ABCH). The *Scarlet* gene in the ABCG subfamily had multiple copies in *H. coronatus* compared to other species, and the *Scarlet* genes of *D. melanogaster*, *D. lobata*, *H. coronatus* and *B. germanica* are indicated by green, blue, red and cyan colors, respectively.

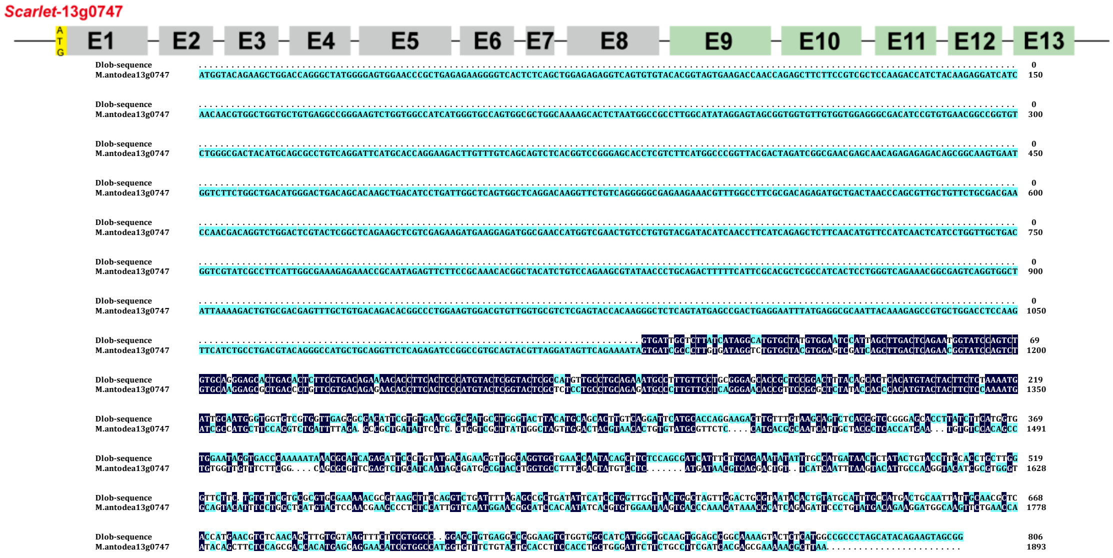

Supplementary Fig. 10 Coding sequence of the *Scarlet* gene (Hcor-13g0747) subjected to blastn against the genome of *D. lobata*. Black shading indicates areas with 100% homology, and cyan shading indicates areas with ≥ 50% identity. The gray squares represent exons missing in *D. lobata,* and the green squares represent residual exons in *D. lobata*.

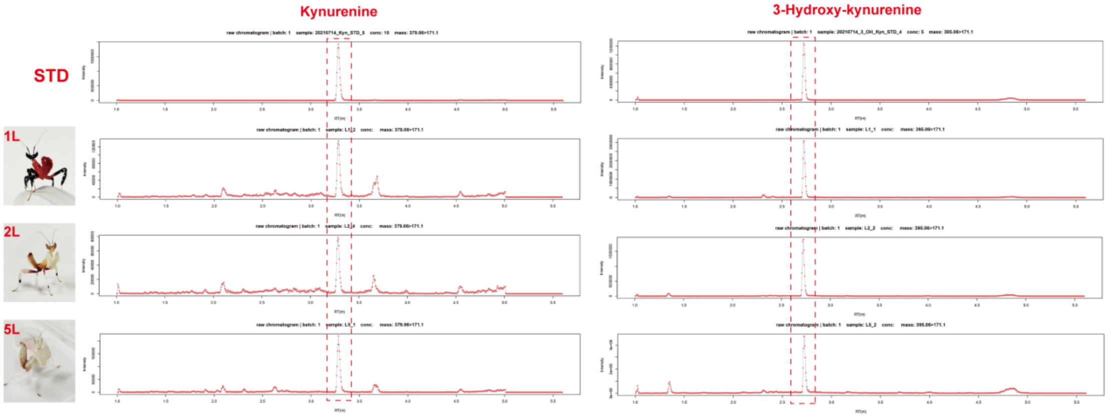

Supplementary Fig. 11 Progressive peaks of kynurenine and 3-hydroxykynurenine generated by ultra-performance liquid chromatography with tandem mass spectrometry (UPLCMS/MS) in the standard (STD) and first (L1), second (L2) and fifth (L5) instars. The red dotted box highlights the time of the peak.

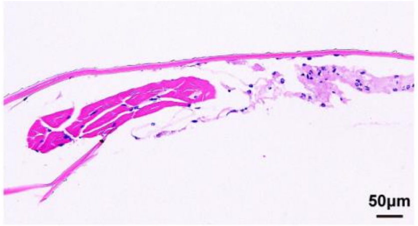

Supplementary Fig. 12 Observation of cell morphology in the *H. coronatus* leg by hematoxylin-eosin staining. Hematoxylin-stained nuclei and eosin-stained cytoplasm. The experiment was repeated twice with similar results.

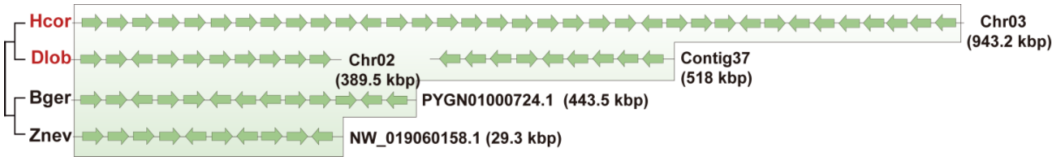

Supplementary Fig. 13 *Cuticle* gene clusters. The arrow indicates each complete gene orientated from 5' to 3'. kbp, kilobase pair. Hcor, *Hymenopus coronatus*. Dlob, *Deroplatys lobata*. Znev, *Zootermopsis nevadensis*. Bger, *Blattella germanica*.

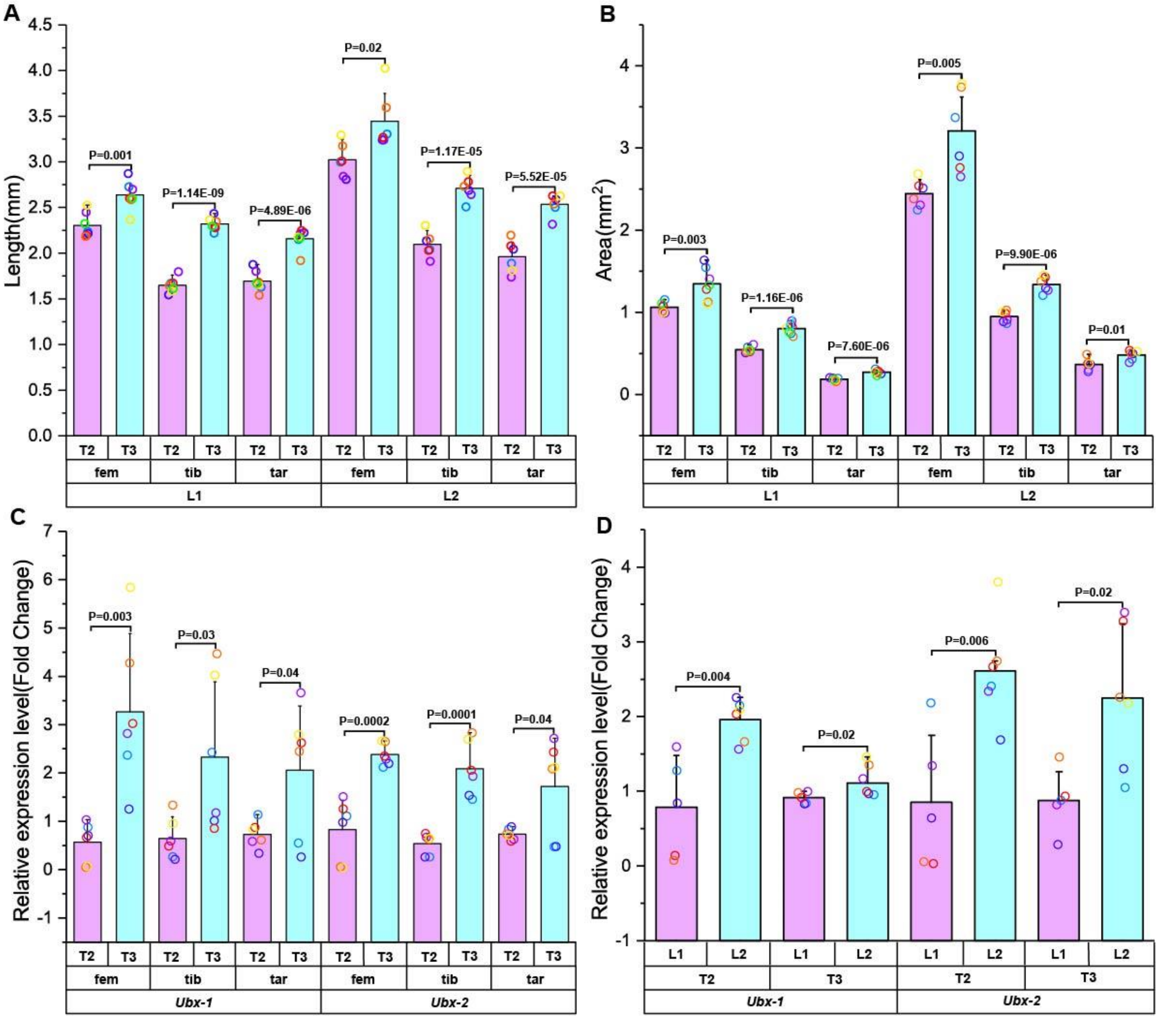

Supplementary Fig. 14 Comparison in size and Ubx expression of T2 and T3 legs. Comparison in length (A) and area (B) of the femur, tibia and tarsus of T2 and T3 legs at L1 and L2 stages. (C) Detection of the expression level of the two transcripts of *Ubx* (*Ubx*-1 and *Ubx*-2) in the femur, tibia and tarsus between T2 and T3 legs at L1 stage. (D) Comparison of the expression level of Ubx in the femur of T2 and T3 legs between L1 and L2 stages. Data represent the mean ± standard deviation. n = 7 per group for A and B, and n = 6 per group for C and D. * $p < 0.05$ and ** $p < 0.01$ by two-sided Student’s *t* tests.

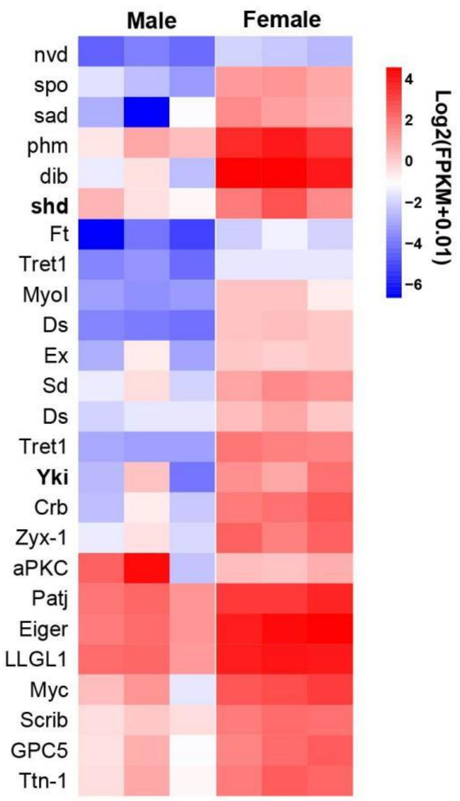

Supplementary Fig. 15 Heatmap of the 25 differential expression genes between females and males.

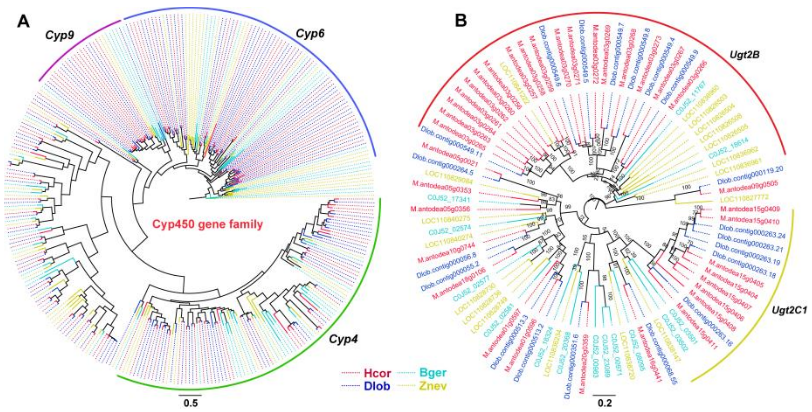

Supplementary Fig. 16 The *CYP450* (A) and *UGT* (B) gene families involved in the metabolism of toxic substances were significantly expanded in the genomes of Hcor and Dlob. The genes of Hcor, Dlob, and Bger are highlighted in red, blue, green, and cyan, respectively. Hcor, *Hymenopus coronatus*. Dlob, *Deroplatys lobata*. Znev, *Zootermopsis nevadensis*. Bger, *Blattella germanica*.

Supplementary Tables

Supplementary Table 1 Chromosome information of *H. coronatus* and *D. lobata.*

H. coronatus		*D. lobata*	
Chr	Length	Chr	Length
LG01	182,135,472.00	LG01	662,812,041.00
LG02	177,328,914.00	LG02	340,671,808.00
LG03	173,556,973.00	LG03	299,055,287.00
LG04	163,751,278.00	LG04	297,316,604.00
LG05	162,945,440.00	LG05	291,307,117.00
LG06	153,951,165.00	LG06	285,040,348.00
LG07	153,775,185.00	LG07	258,975,559.00
LG08	148,745,362.00	LG08	231,107,607.00
LG09	147,347,160.00	LG09	215,223,397.00
LG10	142,118,144.00	LG10	211,914,135.00
LG11	140,564,236.00	LG11	198,621,187.00
LG12	139,836,091.00	LG12	186,782,682.00
LG13	136,106,031.00	LG13	127,469,843.00
LG14	132,510,623.00	LG14	127,342,537.00
LG15	100,147,851.00	Total	3,733,640,152.00
LG16	90,174,698.00		
LG17	88,301,622.00		
LG18	81,284,902.00		
LG19	77,598,682.00		
LG20	76,471,878.00		
LG21	60,673,578.00		
LG22	55,716,183.00		
LG23	41,549,372.00		
Total	2,826,590,840.00		

Supplementary Table 2 Genomic parameters of *H. coronatus* and *D. lobata.*

	H. coronatus	*D. lobata*
Estimated genome size (Gb)	3.18	4.28
Assembly size (Gb)	2.88	3.96
Chromosomes	2n=46	2n=28
Sequencing technology	Nanopore	Nanopore
Coverage	97.91	59.86
Assembler	NextDenovo v2.0-beta.1	NextDenovo v2.0-beta.1
Contig N50 (Mb)	15.76	6.11
Contig N90 (Mb)	2.5	1.07
Number of contigs	840	1853
Scaffold N50 (Mb)	147.35	285.05
Number of scaffolds	160	557
GC content (%)	35.74	37.25
Percentage of repeat sequences	64.94%	66.13%
Number of genes	16,294	17,691
Genes annotated	15,156	15,896
Average gene length (Kb)	77	83.96
Number of transcripts	13,817	15,494
Heterozygosity (%)	0.41	0.6

Supplementary Table 3 Coverage statistics for Illumina and Nanopore sequencing data

		Illumina		Nanopore	
	Depth (×)	Base number	Coverage rate (%)	Base number	Coverage rate (%)
H. coronatus	1	2,872,300,790	99.45	2,887,446,469	99.97
	5	2,867,666,728	99.29	2,885,980,893	99.92
	10	2,864,627,554	99.18	2,884,602,440	99.87
	20	2,859,032,405	98.99	2,881,175,273	99.75
D. lobata	1	3,933,246,040	98.75	3,981,020,748	99.95
	5	3,910,828,248	98.18	3,953,949,138	99.27
	10	3,894,154,084	97.77	3,932,301,455	98.72
	20	3,847,833,155	96.6	3,878,968,547	97.38

Supplementary Table 4 BUSCO assessment of the *H. coronatus* and *D. lobata* genome assemblies.

	H. coronatus		*D. lobata*	
	Number	Percent (%)	Number	Percent (%)
Complete BUSCOs (C)	1603	96.6	1601	96.56
Complete and single-copy BUSCOs (S)	1584	95.5	1551	93.55
Complete and duplicated BUSCOs (D)	19	1.1	50	3.02
Fragmented BUSCOs (F)	33	2	32	1.93
Missing BUSCOs (M)	22	1.4	25	1.51
Total BUSCO groups searched	1658	100	1658	100

Supplementary Table 5 Statistics of repeat prediction in *H. coronatus* and *D. lobata*

H. coronatus				***D. lobata***			
**Class**	**Order**	**Superfamily**	**Percentage of sequence (%)**	**Class**	**Order**	**Superfamily**	**Percentage of sequence (%)**
			22.39				20.79
	LINE		7.64		LINE		11.87
		L2	1.22			L2	0.94
		Penelope	1.25			Unknown	5.04
		I	1.82			Penelope	0.59
		RTE-BovB	0.41			L1-Tx1	0.15
		RTE-X	0.58			R1-LOA	0.5
		R1	0.26			I	2.1
		Jockey	0.89			RTE-BovB	0.5
		CR1	0.21			RTE-X	1
		I-Jockey	0.18			I-Jockey	0.71
		L1-Tx1	0.15	Class I		R1	0.17
		Unknown	0.11			Other	0.17
		LOA	0.11		SINE		2.47
Class I		I-Nimb	0.13			Unknown	1.9
		Other	0.32			MIR	0.43
	LTR		1.2			Other	0.15
		Gypsy	0.53		LTR		6.45
		Pao	0.37			Gypsy	0.98
		Gypsy-Cigr	0.11			Unknown	4.08
		Other	0.18			Ngaro	0.73
	SINE		13.55			ERV1	0.56
		tRNA-V-CR1	0.3			Other	0.11
		tRNA-Deu	9.83				38.77
		tRNA-V	0.95		DNA		25.05
		Unknown	1.7			hAT-Tip100	0.35
		ID	0.49			Kolobok-T2	0.16
		tRNA-I	0.19			Maverick	3.15
		Other	0.09			Unknown	5.48
			19.81			hAT-hAT19	0.45
	DNA		19.39			P	0.12
		TcMar-Tc1	5.39	Class II		PiggyBac	0.26
		TcMar-Mariner	3.33			PIF-Harbinger	0.24
		CMC-EnSpm	4.04			CMC-Chapaev-3	0.19
Class II		Academ-1	0.34			TcMar-m44	0.24
		Unknown	0.2			hAT-Charlie	0.19
		hAT-Tip100	1.33			TcMar-Tc1	11.78
		TcMar-Tc4	0.29			hAT-Blackjack	0.16
		Kolobok-Hydra	0.22			TcMar-Tigger	0.11
		P	0.18			Sola-1	0.2

		TcMar-m44	0.23
		hAT-hAT19	0.11
		TcMar-Tc2	0.14
		hAT-Blackjack	0.18
		Maverick	0.52
		hAT-Ac	0.86
		hAT-Charlie	0.59
		PIF-Harbinger	0.11
		hAT	0.3
		CMC-Chapaev-3	0.14
		PiggyBac	0.15
		Other	0.75
	RC		0.42
		Helitron	0.42
Total TEs			42.2
Unknown			22.38
Simple repeats			0.21
Other			0.15
Low complexity			0.01
Total Repeats			64.94

		TcMar-Mariner	1.23
		Other	0.73
	MITE		7.68
		Unknown	7.68
	RC		6.04
		Helitron	6.04
Total TEs			59.56
Tandem Repeats			0.81
	SSR		0.05
	tandem_repeat		0.76
Unknown			5.62
Simple repeats			0.11
Low complexity			0
Other			0.02
Total Repeats			66.13

Supplementary Table 6 Protein-coding gene annotation statistics for *H. coronatus* and *D. lobata.*

	Gene set	Total number of genes	Average transcript length (bp)	Average CDS length (bp)	Average exon number per gene
H. coronatus	AUGUSTUS	34,610	61,810.70	1,230.21	5.27
	GeMoMa	9,082	54,388.07	1,516.71	7.6
	transdecoder	10,822	63,190.91	1,455.08	6.68
	EVM	16,294	77,169.78	1,542.55	6.92
D. lobata	AUGUSTUS	27,668	82,289.47	1,140.19	5.14
	GeMoMa	35,827	26,404.66	916.92	3.88
	transdecoder	9,981	85,676.05	1,615.22	7.58
	EVM	17,691	83,962.22	1,422.41	6.51

Supplementary Table 7 BUSCO assessment of the *H. coronatus* and *D. lobata* genome annotations.

	H. coronatus		*D. lobata*	
	Number	Percent (%)	Number	Percent (%)
Complete BUSCOs (C)	1,628	98	1,583	95
Complete and single-copy BUSCOs (S)	1,603	97	1,523	92
Complete and duplicated BUSCOs (D)	25	2	60	4
Fragmented BUSCOs (F)	20	1	26	2
Missing BUSCOs (M)	10	1	49	3
Total BUSCO groups searched	1,658	100	1,658	100

Supplementary Table 8 Gene function annotation statistics of *H. coronatus* and *D. lobata.*

		H. coronatus		*D. lobata*	
Type		Number	Percent (%)	Number	Percent (%)
Annotation	Swissprot	12,269	75.30%	12,632	71.40%
	Kegg	7,806	47.91%	7,769	43.91%
	KOG	9,866	60.55%	10,247	57.92%
	GO	8,988	55.16%	7,258	41.03%
	NR	13,674	83.92%	15,287	86.41%
Total	Annotated	15,156	93.02%	15,896	89.85%
	Genes	16,294		17,691	

Supplementary Table 9 Sequencing data volume of Illumina and Nanopore for genome assembly.

	Sample	Reads_number	Bases
Illumina	*H. coronatus*	1072813684	155.56 Gb
	D. lobata	1917898930	286.27 Gb
Nanopore	*H. coronatus*	12674598	297.60 Gb
	D. lobata	14364636	258.05 Gb

Supplementary Table 10 Primers for real-time quantitative polymerase chain reaction and siRNA for RNA interference.

Gene	Sequence (5' to 3')
Gapdh-F	TGCACCCATGTTTGTGGTTG
Gapdh-R	ACCATCCACAGTTTTCTGCGT
Arm-F	CTTGGCCATCCAAGTCAACG
Arm-R	ACAGAATACCAGCAGCGCAA
Ubx-1-F	TCTCCATACGCCTCTACCCA
Ubx-1-R	AAGAACCCGTCGTGCTGTAG
Ubx-2-F	GAAGAGGCAGACAGACGTACA
Ubx-2-R	CTTGATTTGCCGCTCCGTGA
Arm-sense	CAGAAUUGUUUGUGGACAUTT
Arm-antisense	AUGUCCACAAACAAUUCUGTT
