# Supplementary material for: Evolutionary genomics of camouflage innovation in the orchid mantis
Source: Nat Commun. 2023 Aug 10;14:4821. doi: 10.1038/s41467-023-40355-1 (PMC10415354; doi:10.1038/s41467-023-40355-1)
Supplement: Supplementary file 3 — Reporting Summary [file 41467_2023_40355_MOESM3_ESM.pdf]

nature portfolio

Corresponding author(s): Fuwen Wei

Last updated by author(s): Jul 12, 2023

Reporting Summary

Nature Portfolio wishes to improve the reproducibility of the work that we publish. This form provides structure for consistency and transparency in reporting. For further information on Nature Portfolio policies, see our Editorial Policies and the Editorial Policy Checklist.

Statistics

For all statistical analyses, confirm that the following items are present in the figure legend, table legend, main text, or Methods section.

n/a	Confirmed	
☐	☒	The exact sample size (n) for each experimental group/condition, given as a discrete number and unit of measurement
☐	☒	A statement on whether measurements were taken from distinct samples or whether the same sample was measured repeatedly
☐	☒	The statistical test(s) used AND whether they are one- or two-sided<br>*Only common tests should be described solely by name; describe more complex techniques in the Methods section.*
☒	☐	A description of all covariates tested
☐	☒	A description of any assumptions or corrections, such as tests of normality and adjustment for multiple comparisons
☒	☐	A full description of the statistical parameters including central tendency (e.g. means) or other basic estimates (e.g. regression coefficient) AND variation (e.g. standard deviation) or associated estimates of uncertainty (e.g. confidence intervals)
☐	☒	For null hypothesis testing, the test statistic (e.g. F, t, r) with confidence intervals, effect sizes, degrees of freedom and P value noted<br>*Give P values as exact values whenever suitable.*
☒	☐	For Bayesian analysis, information on the choice of priors and Markov chain Monte Carlo settings
☒	☐	For hierarchical and complex designs, identification of the appropriate level for tests and full reporting of outcomes
☒	☐	Estimates of effect sizes (e.g. Cohen's d, Pearson's r), indicating how they were calculated

Our web collection on statistics for biologists contains articles on many of the points above.

Software and code

Policy information about availability of computer code

Data collection

Short-reads sequencing data were generated by Illumina HiSeq X-Ten (San Diego, USA).
Long-reads sequencing data were generated by Nanopore PromethION (Oxford Nanopore Technologies, UK).
Hi-C sequencing data were generated by Illumina HiSeq 2500 platform (San Diego, USA).
The ultrastructure of legs was captured by a JEM-1200EX transmission electron microscope (TEM, JEOL, Japan)
The length and area of the femur, tibia and tarsus of T2 and T3 legs were measured by ImageJ2 (http://imagej.net/).
Quantitative real-time PCR was performed using Mx3000P Real-Time PCR System (Agilent Technologies, Inc.).
The concentrations of kynurenine and 3-hydroxykynurenine were determined by HPLC (Waters Corp., Milford, MA, USA).

Data analysis

A combination of Nanopore sequencing, Illumina sequencing and Hi-C sequencing was used to generate mantis genome assemblies. Contigs were assembled by NextDenovo software (v2.0-beta.1, https://github.com/Nextomics/NextDenovo), and the assembled contig-level genomes were polished by NextPolish (v1.0.5) and Pilon (v1.22). Then, the contigs were anchored into chromosomes by Hi-C sequencing reads through the Juicer (v1.5) and 3D-DNA (v180922) software workflow. To further improve the chromosome-scale assembly, it was subjected to manual review and refinement using Juicebox Assembly Tools (https://github.com/theaidenlab/juicebox). Finally, genome quality was estimated with BUSCO (insecta_odb9, v3.0.2), k-mer analysis, and by mapping back the initial reads to the assembly.

Transposable elements were identified using RepeatMasker (open-4.0.7), RepeatModeler (v1.0.8), and MITE-Hunter (v1.0.8). Gene structures were determined by combining ab initio and homology methods. For ab initio annotation, we used Augustus (v3.2.1) and GENSCAN (v1.0) to analyze the repeat-masked genome. For homolog-based annotation, protein sequences of fruit fly (Drosophila melanogaster), cockroach (Blattella germanica), honeybee (Apis mellifera), mosquito (Aedes aegypti), and small brown planthopper (Laodelphax striatella) genomes were aligned to mantis genome sequences using BLAST software (v2.3.0). Together with transcriptomic data, gene sets from these three methods were then integrated by EVidenceModeler software (v1.1.1). For gene functional annotation, the integrated gene set was

aligned against public databases, including KEGG, Swiss-Prot, TrEMBL, COG, and NR with BLAST (v2.3.0), and merged with annotations by InterProScan (v4.8) software. The integrity of annotation was estimated by comparison with reference genome annotations and BUSCO (v3.0.2), resulting in 95.5%–98.2% completeness according to BUSCO analysis, suggesting the high quality of the annotation.

To cluster families of protein-coding genes, we extracted protein sequences from the genomes of H. coronatus, D. lobata, and 16 other species of Insecta. The protein sequences were aligned reciprocally (i.e. all-vs.-all) using BLASTP programs with an E-value ≤1e−5, and then clustered using orthoMCL (v2.0.9). RAxML (v8.2.10) was used to construct a phylogenetic tree for the super-alignment using the GTRGAMMA model. The MCMCTree program of the PAML (v4.8) package was used to determine divergence times with the approximate likelihood calculation method and three dated fossil records.

Family expansion or contraction analysis was performed by CAFÉ (v3.1) calculations with the parameters lambda -s and p < 0.01 based on the phylogenetic tree constructed above. To trace the demographic history of H. coronatus and D. lobata, we employed PSMC to estimate changes in effective population size using heterozygous sites, with the following set of parameters: –N 30 –t 15 –r 5 –p 4 + 25×2 + 4 + 6. Genome-wide protein sequences of H. coronatus, D. lobata, Z. nevadensis, B. germanica, and D. melanogaster were extracted, and the conserved nucleotide binding domain (PF00005.24) and transmembrane domain (PF00664.20) were scanned genome-wide for candidate ABC transporter genes using the Hidden Markov Model (HMM) in R (v3.2.1). To assign the candidate ABC genes into different subfamilies, multiple alignments of the ABC transporter protein sequences were performed using MUSCLE (v3.8.31), and the poorly aligned regions and partial gaps were removed with trimAl (gt = 0.5). Then, the alignments were subjected to phylogenetic analysis using RAxML based on the Maximum Likelihood method with parameters: -f a -x 12345 -N 1000 -p 12345 -m PROTGAMMAWAG". The resulting trees were displayed and edited using FigTree (v1.4.4, https://github.com/rambaut/figtree/releases).In addition, the same analyses were performed to identify Cuticle (pfam:PF00379), Trypsin (pfam:PF00089), UGT (pfam:PF00201), and CYP450 (pfam:PF00067).

For manuscripts utilizing custom algorithms or software that are central to the research but not yet described in published literature, software must be made available to editors and reviewers. We strongly encourage code deposition in a community repository (e.g. GitHub). See the Nature Portfolio guidelines for submitting code & software for further information.

Data

Policy information about availability of data

All manuscripts must include a data availability statement. This statement should provide the following information, where applicable:

- Accession codes, unique identifiers, or web links for publicly available datasets
- A description of any restrictions on data availability
- For clinical datasets or third party data, please ensure that the statement adheres to our policy

The sequence data and the genome assemblies have been deposited to the National Genomics Data Center, China (https://ngdc.cncb.ac.cn/), with accession number CRA010804 (https://bigd.big.ac.cn/gsa/browse/CRA010804).

Human research participants

Policy information about studies involving human research participants and Sex and Gender in Research.

Reporting on sex and gender	not applicable
Population characteristics	not applicable
Recruitment	not applicable
Ethics oversight	not applicable

Note that full information on the approval of the study protocol must also be provided in the manuscript.

Field-specific reporting

Please select the one below that is the best fit for your research. If you are not sure, read the appropriate sections before making your selection.

☒ Life sciences ☐ Behavioural & social sciences ☐ Ecological, evolutionary & environmental sciences

For a reference copy of the document with all sections, see nature.com/documents/nr-reporting-summary-flat.pdf

Life sciences study design

All studies must disclose on these points even when the disclosure is negative.

Sample size	In this study, for RNA-seq and functional experiments, more than 3 individuals was chosen, which are generally sufficient for statistical test. The precise sample sizes have been provided in the Methods section and figure legends.
Data exclusions	No data were excluded from the analyses.
Replication	All the data were collected by 3 individual experiments. All attempts at replication were successful.
Randomization	The RNA-seq and functional experiments were performed based on different developmental stages of Hymenopus coronatus. For data

	analysis, more than 3 individuals at each developmental stage were chosen randomly.
Blinding	All the experiments were performed by the investigators that were blinded to group allocations.

Reporting for specific materials, systems and methods

We require information from authors about some types of materials, experimental systems and methods used in many studies. Here, indicate whether each material, system or method listed is relevant to your study. If you are not sure if a list item applies to your research, read the appropriate section before selecting a response.

Materials & experimental systems

n/a	Involved in the study
☒	☐ Antibodies
☒	☐ Eukaryotic cell lines
☒	☐ Palaeontology and archaeology
☐	☒ Animals and other organisms
☒	☐ Clinical data
☒	☐ Dual use research of concern

Methods

n/a	Involved in the study
☒	☐ ChIP-seq
☒	☐ Flow cytometry
☒	☐ MRI-based neuroimaging

Animals and other research organisms

Policy information about studies involving animals; ARRIVE guidelines recommended for reporting animal research, and Sex and Gender in Research

Laboratory animals	Captive breeding individuals of Hymenopus coronatus hatched from the same ootheca that was collected from the Xishuangbanna rainforest, Yunnan Province, China in 2018. Individuals of Deroplatys lobata were collected from a captive breeding center in Beijing, China in 2018. All sample collections have been approved by the Institutional Animal Care and Use Committee of the Institute of Zoology, Chinese Academy of Sciences. All individuals were housed in semitransparent cages (7 cm × 7 cm × 9 cm) in an incubator with a temperature range of 26–27°C, 80% relative humidity, and a 16 h/8 h light/dark photoperiod. The tissue samples of each developmental stage including L1, L2, L5 and adult were collected respectively.
Wild animals	There is no wild animals used in the study.
Reporting on sex	This finding do not apply to only one sex.
Field-collected samples	No field-collected samples is used in this study.
Ethics oversight	All animal experimental procedures were approved by the Institutional Animal Care and Use Committee of the Institute of Zoology, Chinese Academy of Sciences. We have complied with all relevant ethical regulations for animal research.

Note that full information on the approval of the study protocol must also be provided in the manuscript.
